# Survivorship of the dual-mobility construct in elective primary total hip replacement: a systematic review and meta-analysis including registry data

**DOI:** 10.1007/s00402-023-04803-3

**Published:** 2023-02-17

**Authors:** Andrew Gardner, Hamish Macdonald, Jonathan T. Evans, Adrian Sayers, Michael R. Whitehouse

**Affiliations:** 1 Translational Health Sciences, University of Bristol, Bristol, UK; 2 Musculoskeletal Research Unit, Translational Health Sciences, Bristol Medical School, Southmead Hospital, Bristol, UK

**Keywords:** Total hip replacement, Dual mobility

## Abstract

**Introduction:**

Dislocation is a common complication associated with total hip replacement (THR). Dual-mobility constructs (DMC-THR) may be used in high-risk patients and have design features that may reduce the risk of dislocation. We aimed to report overall pooled estimates of all-cause construct survival for elective primary DMC-THR. Secondary outcomes included unadjusted dislocation rate, revision for instability, infection and fracture.

**Methods:**

MEDLINE, EMBASE, Web of Science, Cochrane Library and National Joint Registry reports were systematically searched (CRD42020189664). Studies reporting revision (all-cause) survival estimates and confidence intervals by brand and construct including DMC bearings were included. A meta-analysis was performed weighting series by the standard error.

**Results:**

Thirty-seven studies reporting 39 case series were identified; nine (10,494 DMC-THR) were included. Fourteen series (23,020 DMC-THR) from five national registries were included.

Pooled case series data for all-cause construct survival was 99.7% (95% CI 99.5–100) at 5 years, 95.7% (95% CI 94.9–96.5) at 10 years, 96.1% (95% CI 91.8–100) at 15 years and 77% (95% CI 74.4–82.0) at 20 years. Pooled joint registry data showed an all-cause construct survivorship of 97.8% (95% CI 97.3–98.4) at 5 years and 96.3% (95% CI 95.6–96.9) at 10 years.

**Conclusions:**

Survivorship of DMC-THR in primary THR is acceptable according to the national revision benchmark published by National Institute for Clinical Excellence (NICE).

**Supplementary Information:**

The online version contains supplementary material available at 10.1007/s00402-023-04803-3.

## Introduction

Total hip replacement (THR) is common and successful [1]. Dislocation is a recognised complication, more than half occur within 3 months of primary surgery [2]. The incidence of dislocation following primary THR ranges from 0.12 to 16.13% at an average follow-up of 6 years [3]. In the National Joint Registry (NJR), dislocation or subluxation was the second most common reason for the first revision (17.4%), contributing to 361.3 revisions annually [4]. Interventions not requiring any change of implants are not captured by the NJR.

Risk factors associated with dislocation can be categorised into patient, surgical, implant and hospital related [3]. Patient-related factors include older age, high body mass index, drug use disorders, social deprivation, low income, neurological and rheumatoid disorders, increasing comorbidity indices and previous spine or hip surgery. Surgical factors include surgical approach.

To mitigate the risk of dislocation, the use of large diameter femoral heads has grown in popularity, and according to the NJR, the two most frequently used head sizes in 2020 were 32 mm and 36 mm [4]. Several studies have observed an association between larger head size and a lower dislocation rate [5, 6]. There is, however, an association between 36-mm heads and higher revision rates [4]. This observation may be explained by the proportional relationship between wear volume and sliding distance [7]. Other solutions for instability include constrained liners and may be more appropriate in the setting of complex revision [8]. An alternative is the use of a dual-mobility construct (DMC-THR). DMC-THRs were developed in the 1970s to increase the range of motion before prosthetic impingement and to increase the jump distance before dislocation [7]. DMC-THRs utilise two articulations, one between the head and polyethylene (PE) liner and another between the PE head and acetabular shell [9]. The mobile PE liner acts as a large diameter head increasing head/neck ratio, jump distance and arc of motion before prosthetic impingement. Intra-prosthetic dislocation (IAPD) is a unique complication of the DMC-THR where the head dislodges from the mobile PE component [10]. The increased sliding distance and the second bearing surface increase frictional torque which may increase wear, osteolysis and loosening [7]. This mechanism may also lead to an increased risk of periprosthetic fracture (PPF) and metallosis [11]. Despite these issues, in the USA, the use of DMC-THR has doubled in the last decade, comprising 12% of all primary THRs in 2018 [12].

A recent review article, summarising 24 case series (10,783 DMC-THRs), reported a mean survivorship of 98% (83.8–100%) at a mean follow-up of 8.5 (1.8–16.5) years [13]. Several systematic reviews have shown an association between DMC-THRs and lower dislocation rates when compared to a conventional total hip replacement (C-THR) [14–16]. Such studies are, however, susceptible to selection and publication bias and may overestimate survival [17]. These problems can be overcome by looking at national joint registries which include the entire population as its sample, making results more generalisable. In the UK, the National Institute for Clinical Excellence (NICE) and Orthopaedic Data Evaluation Panel (ODEP) recommend that THR revision rates should be 5% or lower at 10 years [18, 19].

The purpose of this review is to synthesise pooled estimates of all-cause construct survival after primary DMC-THR with the inclusion of National Joint Registry data, something that has not been done previously. In addition, we aimed to synthesise estimates of unadjusted dislocation rate (when available) and revision for instability, infection and fracture.

## Methods

### Search strategy and paper selection

The study was registered with the prospective register of systematic reviews, PROSPERO (CRD42020189664), and carried out in accordance with PRISMA guidelines [20]. Systematic searches were conducted in MEDLINE, EMBASE, Web of Science and the Cochrane Library from inception to October 2021 using OVID Silver Platter. Reference lists of included articles and bibliographies of systematic reviews were searched for additional studies. The electronic search strategy combined free and MeSH search terms related to population (e.g. “primary total hip replacement”), intervention (e.g. “dual-mobility cup”) and outcome (e.g. “dislocation”, “instability”, “revision”) (Appendix 1). The website of the International Society of Arthroplasty Registries (ISAR) was checked for a list of its members, and their most recent annual reports were scrutinised for stated outcomes to capture national joint replacement registry data.

All titles and abstracts of studies retrieved from the databases were screened. Papers were filtered by primary author (AG) using Rayyan [21]. Full texts were checked for eligibility by two independent authors (AG and HM). Papers were eligible if they were longitudinal studies, cohort, case cohort, case series or clinical trials. National Joint Registry data that reported revision as an outcome for DMC-THR were included. When specified, we treated each brand construct reported within registries as a different series. When series were not reported by brand, we included series by fixation and bearing construct. Case reports, conference abstracts, surgical technique descriptions, review articles and animal trials were excluded. Papers that included patients receiving a DMC-THR for proximal femoral fractures or revision surgery were excluded because they represent a different population with different risk and survival profile [22].

### Data extraction and patients

The population included all patients undergoing elective primary THR with DMC bearing. All-cause revision of any part of the construct was the primary outcome. A revision was defined as per the NJR as “any operation performed to add, remove or modify one or more component” [4]. The secondary outcomes were unadjusted dislocation rate and revision for instability, infection and fracture.

Descriptive and quantitative information was extracted into a standardised Excel spreadsheet (version 16.45, Microsoft, USA). Data were extracted on publication date, study design, patient and implant characteristics, survival estimates at any time point, number of dislocations and number of revisions for instability, infection and fracture. When case series were published in multiple papers, the most recent report was used.

### Statistical analysis

Statistical analysis was conducted using Stata IC (version 16.1, StataCorp, USA). Survival estimates were pooled with meta-analysis weighting each series on the overall pooled estimate according to its standard error (calculated from published confidence intervals). Weighted means were calculated for continuous variables. When studies did not report survival at exact time points, figures were rounded up to the nearest 5 years as a separate sensitivity analysis.

### Risk-of-bias assessment

Study quality was assessed using a non-summative scoring system described by Wylde et al. [23].

## Results

**Fig. 1:**
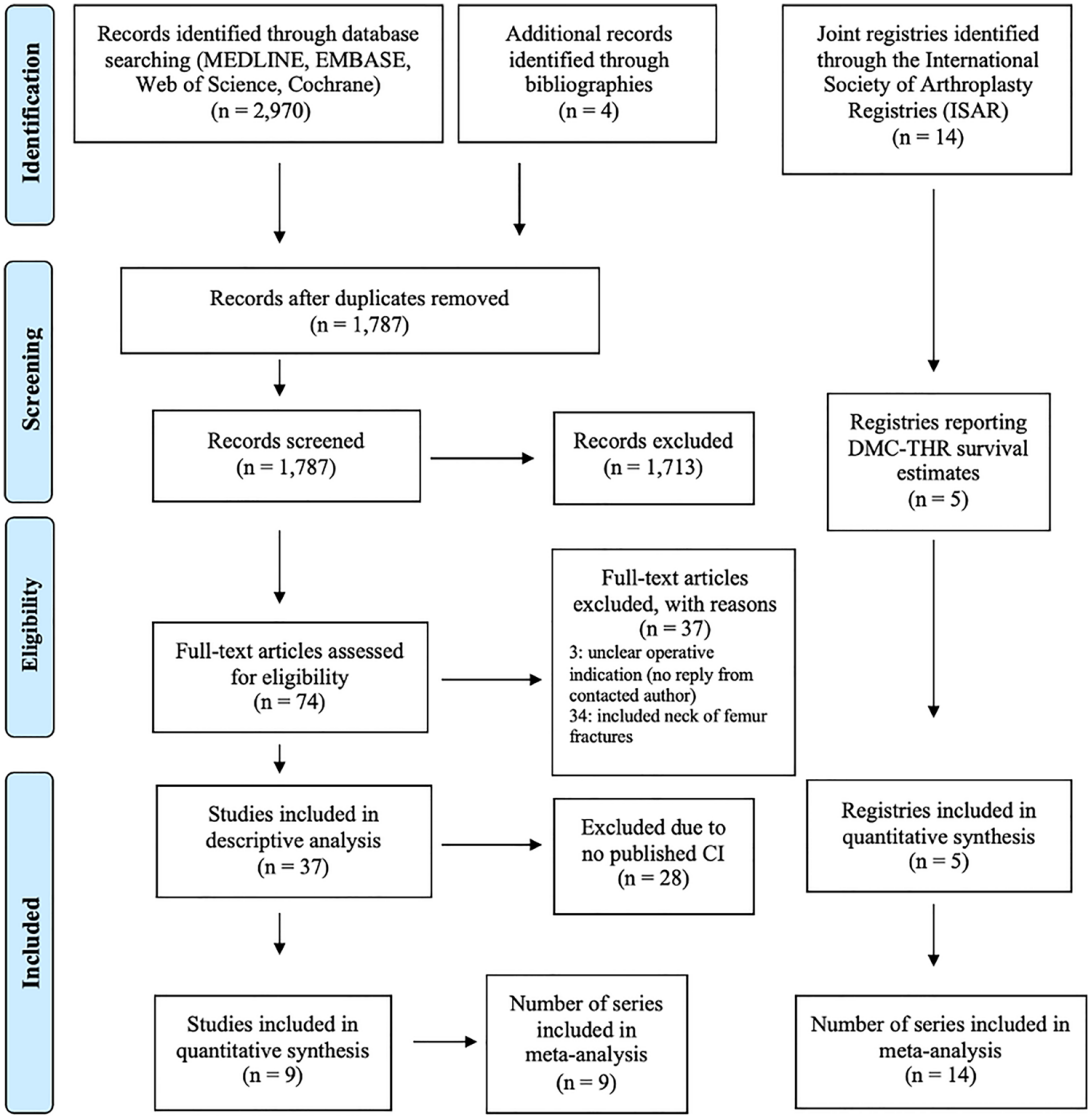
PRISMA flow diagram of searches and included studies

The search produced 2970 references and four additional citations through manual reference lists searches. After de-duplication, there were 1787 articles screened leaving 74 full texts for review. Thirty-seven were excluded, leaving 37 articles, reporting 39 cases series. Of these, 14 of 37 reported all-cause construct survival of which nine published confidence intervals (Fig. 1). Table 1 provides a summary of patient characteristics (individual studies: appendix 2). Of the 14 registries identified through ISAR, five published DMC-THR survival estimates and provided 14 individual brand and construct-based series.

**Table 1:** Characteristic of contributing data sources

	Individual case series	National Joint Registry. Annual report 2021	Australian Orthopaedic Association National Joint Replacement Registry. Annual report 2021	Swiss National Joint Registry. Annual report 2020	The Dutch Arthroplasty Registry. Annual report 2020	The German Arthroplasty Registry. Annual report 2020
Study-level characteristics						
Location	3 countries	UK	Australia	Switzerland	The Netherlands	Germany
Number of series	9	5	1	3	3	2
Year of publication	2011–2020	2021	2021	2020	2020	2021
Participant-level characteristics						
Total joint replacements (dual-mobility cups) included	10,494	7569	10,763	1900	1355	1433
Mean age (years)	70.30*	> 65^+^	NR	> 65^+^	NR	NR
Number of females (%)	6101 (58.9)	NR	NR	NR	NR	NR

NR: not reported, +: all patients > 65

*Weighted mean for age by number in study

### Case series

**Fig. 2:**
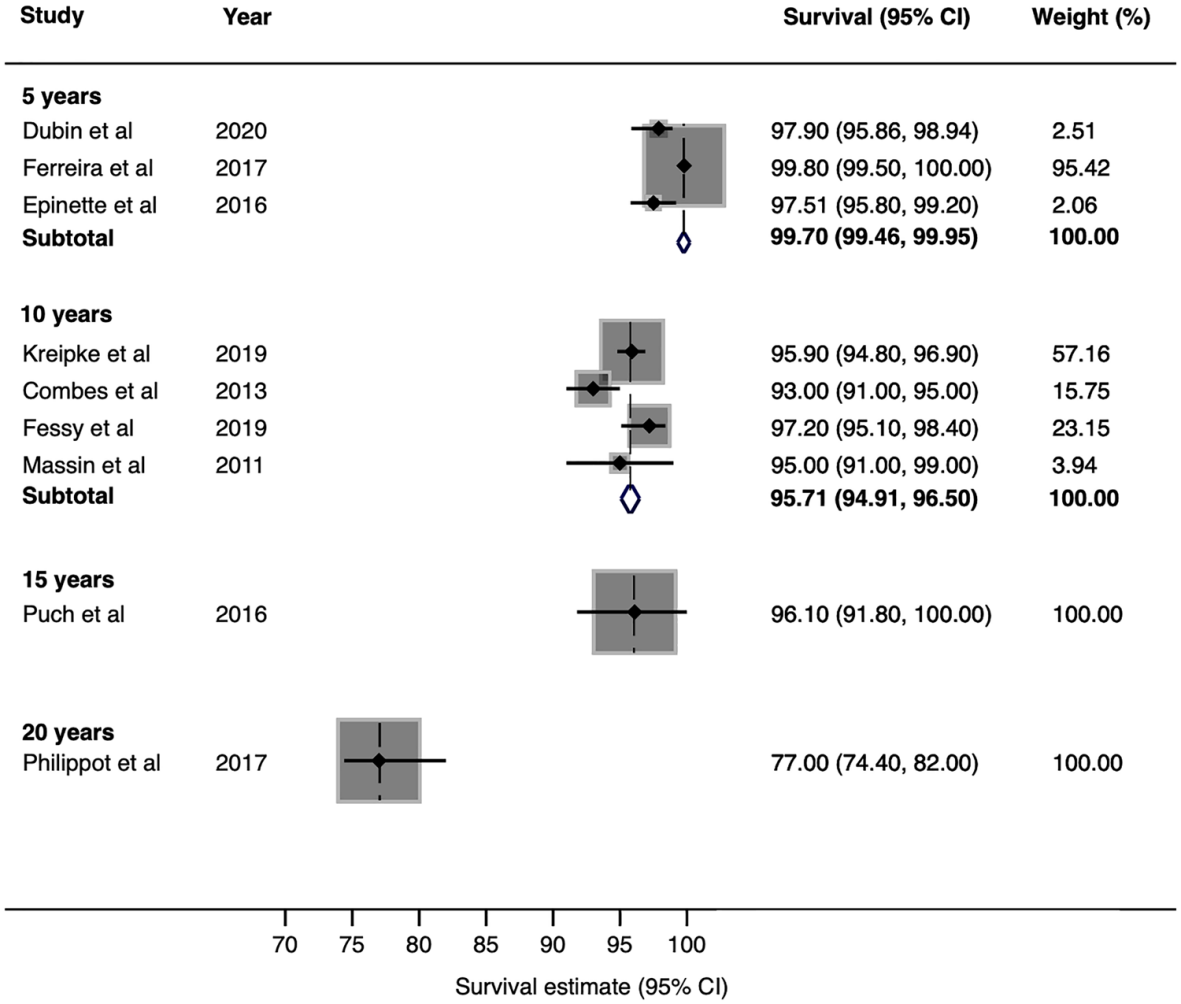
Estimates of survival from case series at 5 years, 10 years, 15 years and 20 years

The nine case series included in the meta-analysis reported all-cause construct survival in 10,494 DMC-THR (range 119–3474) with a follow-up ranging from 4 to 20 years. Pooled analysis of data derived from case series reported at exactly 5, 10 and 20 years showed all-cause survivorship of the DMC-THR of 97.5% (95% CI 95.8–99.2) at 5 years, 95.5% (95% CI 94.3–96.7) at 10 years and 77% (95% CI 73.2–80.8) at 20 years. After rounding, pooled analysis of data extracted from case series of DMC-THR we observed a pooled all-cause construct survival of 99.7% (95% CI 99.5–100) at 5 years, 95.7% (95% CI 94.9–96.5) at 10 years, 96.1% (95% CI 91.8–100) at 15 years and 77% (95% CI 7.4–82.0) at 20 years (Fig. 2).

The overall rate of dislocation, including closed reductions and revision, reported in the 39 case series (17,135 DMC-THR) was 1.1% with a mean patient age at the time of intervention to treat the dislocation of 66.5 years (weighted) at a mean follow-up of 7.3 years (2–25.3). The proportion of females was 60.8%. The overall revision estimate for DMC-THR instability, infection and fracture was 0.8%, 0.4% and 0.3%, respectively (individual studies: appendix 3).

The quality of included case series was variable. The quality assessment showed that two (22.2%) out of nine were consecutive, seven (77.8%) were multicentre, five (55.6%) had less than 80% follow-up and two (22.2%) used multivariable analysis.

### Registry series

**Fig. 3:**
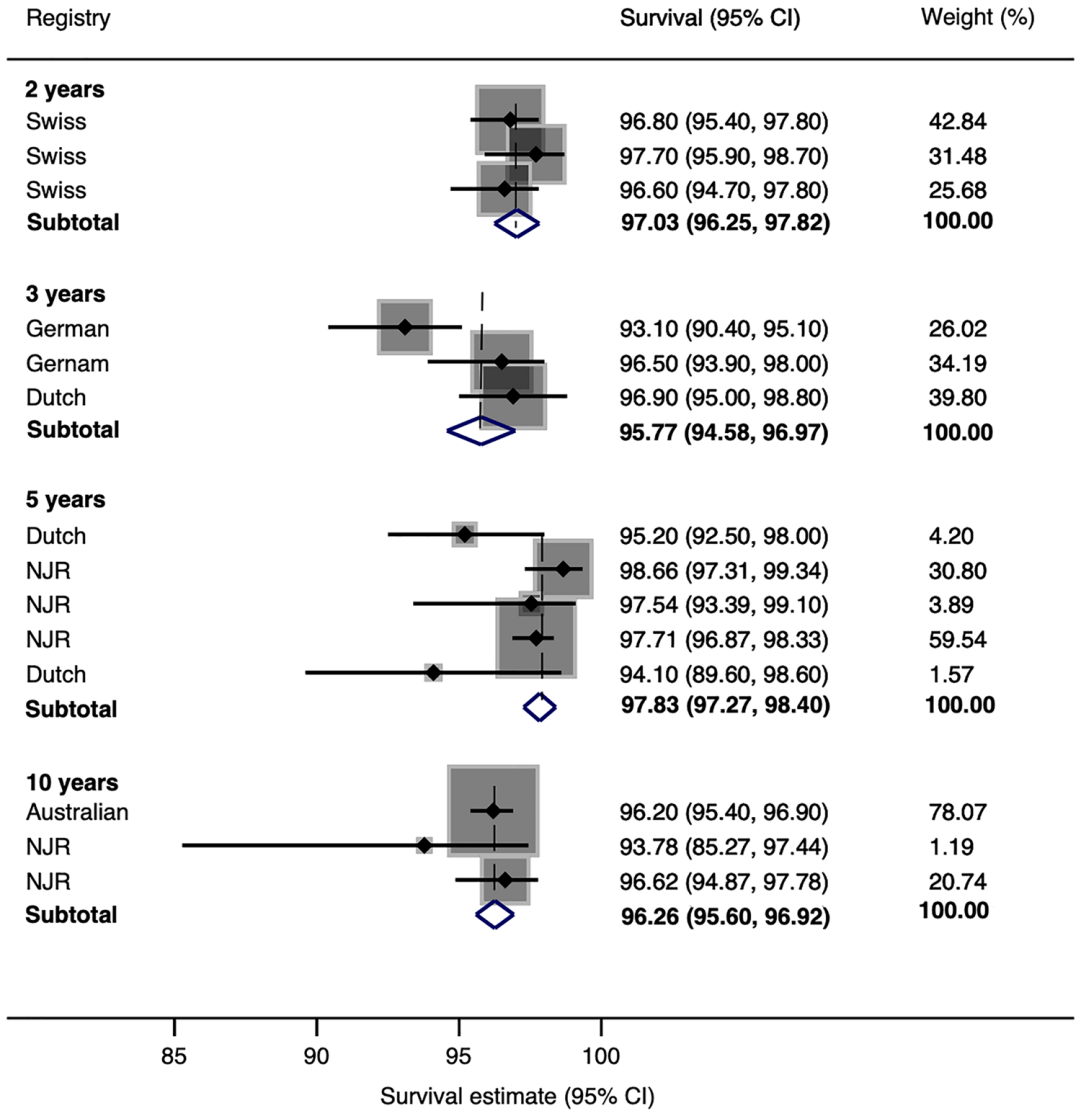
Estimates of survival from registries at 2 years, 3 years, 5 years and 10 years

The search of joint registries revealed 14 brand and construct-based series, all of which provided confidence intervals in 23,020 (range 347–10,763) DMC-THR. Pooled analysis of data extracted from joint registries of DMC-THR showed all-cause construct survival of 97.0% (95% CI 96.3–97.8) at 2 years, 95.8 (95% CI 94.6–97.0) at 3 years, 97.8% (95% CI 97.3–98.4) at 5 years and 96.3% (95% CI 95.6–96.9) at 10 years (Fig. 3).

## Discussion

The pooled survival estimate for DMC-THR at 5 years was 97.8% from registry data and 99.7% from case series. The pooled survival estimate at 10 years was 96.3% from registry data and 95.7% from case series. Survival estimates at 15 and 20 years relied on case series data and were 96.1% and 77%. The unadjusted rate of DMC-THR dislocation was 1.1%. The revision estimate for DMC-THR instability, infection and fracture was 0.8%, 0.4% and 0.3%, respectively. At comparable time points, the survival estimate of DMC-THRs from case series was superior at 5 years but similar at 10 years when compared to registry series. The survival estimate of DMC-THRs at 20 years was from one case series that reported on first-generation DMC-THRs which may account for the apparent drop in survival after this time point.

The results presented are different to previously published survival estimates of primary DMC-THR. One systematic review published in 2018 reported a mean survival of DMC-THR of 98% at a mean follow-up of 8.5 years [13]. However, these figures were based on case series and survival estimates that did not include all-cause revision as an outcome. This means that the results are prone to selection and publication bias and are based on an outcome that may not be relevant to patients. These sources of bias may explain the higher survival estimates seen in case series at 5 years [17]. Jonker et al. reported all-cause revision estimates of 1.6% from case series at a minimum follow-up of 0.5–10 years and 2.7% from registry studies at a median follow-up of 2.5–3.2 years [24]. Despite the inclusion of hip fractures, this supports our theory that case series may overestimate survival and that registry data are more representative of the entire population at risk. Our study also includes much larger numbers of DMC-THR and longer follow-up times than these studies. Despite the higher revision estimates reported in this study, the estimates still fall within the acceptable thresholds set out by NICE. The observed pooled dislocation rate of our study was lower than other published estimates. Darrith et al. reported a rate of 1.5% at a mean follow-up of 8.5 years in a population that included neck of femur fractures [13]. De Martino et al. reported a dislocation rate of 0.9%, excluding IAPD, at a mean follow-up of 6.8 years which may be more representative of contemporary DMC-THR [15, 25]. A meta-analysis published in 2019 of eight comparative non-randomised studies reported that DMC-THR appears to reduce the rate of dislocation [16]. If we assume that there is selective use of DMC-THR for patients at a higher risk of dislocation, the survival estimates for dislocation and revision for dislocation observed to be the same between DMC-THR and C-THR imply that DMC-THR may be beneficial in moderating the higher risk of dislocation. However, reducing the rate of one complication may not warrant its use when there is an association with other complications such as infection and PPF [4, 26]. In addition, the cost of DMC-THR can be up to double that of a C-THR [27]. Its routine use, therefore, may not be justified if survival estimates are comparable to C-THR or potentially worse for other causes of failure.

There is a paucity of randomised comparative trials in this area and designing such a study to provide evidence of causation is difficult due to cost, the large sample sizes required and the challenges of long-term follow-up of joint replacement [28]. One meta-analysis of five comparative studies reported no difference in the all-cause risk of revision between DMC-THR and C-THR [14]. However, only one study was prospective and none were randomised. A proposed nested registry trial may go some way in providing higher-quality evidence [29]. However, concerns have been raised about the trial’s generalizability because several patient groups that are generally regarded as high risk for dislocation are to be excluded. The study may also be underpowered as the power calculation was based on studies that included such patients [30].

The results of this study must be interpreted in the context of its limitations. A total of 6,315 DMC-THRs had to be excluded from the quantitative analysis because the authors did not provide all-cause DMC-THR survival estimates or confidence intervals [31–35]. Some authors chose to publish survival estimates for specific end points: aseptic cup loosening only [36–41], all-cause cup failure only [42] or cup failure after removing patients who were revised for sepsis [43]. Reporting survival of part of the DMC-THR does not match the lived patient experience or patient preference for defining revision outcomes of THR and may bias any conclusion made, falsely suggesting a positive association between DMC-THR and superior survival estimates. All-cause construct survival is closer to what is an acceptable outcome for patients and is recommended by ISAR as the principal outcome measure for both early and late benchmarking [44]. Reporting survival of an implant only is, in itself, also a limitation [45]. Qualitative studies have shown that function and being able to engage in valued everyday activities matters most to patients [46]. Most studies included in this review were retrospective case series and are exposed to confounding and bias. For example, a proportion of patients such as those who are older with multiple comorbidities may be less likely to be offered revision surgery. This also highlights a limitation of NJR data in that it does not report on closed reductions, PFF treated with interventions that do not involve revision and infections that are not revised. These factors may bias the outcome away from the null hypothesis. Only two studies included in the final analysis adjusted their results for any potential confounders which may lead to a conclusion of a false association between DMC-THR and lower dislocations rates [37, 47]. A strength of our study is the inclusion of 23,020 DMC-THRs from national registries, which reduces one source of bias and may better reflect survival in the general population. In addition, it is also the largest study of survival estimates of primary DMC-THRs.

The results in our study suggest that selective use of DMC-THR in primary THR may be justified to reduce the risk of dislocation. However, increased costs and other causes of failure must be taken into consideration with its use. In-depth scrutiny of generalizable early warnings will be paramount to mitigate against potentially higher rates of early revision surgery.

In conclusion, pooled survival estimates of the DMC-THR in primary THR at 5 and 10 years reported in this study are acceptable according to the revision threshold set out by NICE but its use should be carefully considered in light of its cost and outcomes.

## Supplementary Information

Below is the link to the electronic supplementary material.

Supplementary file1 (DOCX 29 KB)

## Data Availability

The authors declare that the data supporting the findings of this study are available within the article (and its supplementary information files).
