# Supplementary material for: Survivorship of the dual-mobility construct in elective primary total hip replacement: a systematic review and meta-analysis including registry data
Source: Arch Orthop Trauma Surg. 2023 Feb 17;143(9):5927–34. doi: 10.1007/s00402-023-04803-3 (PMC10449688; doi:10.1007/s00402-023-04803-3)
Supplement: Supplementary file 1 — Supplementary file1 (DOCX 29 KB) [file 402_2023_4803_MOESM1_ESM.docx]

**Appendix 1. Search strategy**

| **Hip arthroplasty** | **1** | exp arthroplasty, replacement, hip/ |
| --- | --- | --- |
|  | **2** | exp hip prosthesis/ |
|  | **3** | ((primary* or elective* or total*) AND hip* AND (replacement* or arthroplasty or prosthe*)) or ((hip*) and (replacement* or arthroplasty or prosthe*)) or THR or THA.mp |
|  | **4** | 1 OR 2 OR 3 |
| **Dual mobility** | **5** | (dual* or double* or tri*) AND (mobility or articulation* or polar or bearing*).mp |
|  | **6** | (Lima Delta* or MDM* or Mathays* or HipNgo* or Versafit* or JRI ACE or Novae* or Avantage or Saturne* or Fixa Poly or Polarcup or SeleXys or Cupule Quattro or Cupule HAP or Evora or ADM).mp |
|  | **7** | 5 OR 6 |
| **Outcome** | **8** | exp hip dislocation/ |
|  | **9** | exp joint instability/ |
|  | **10** | (dislocation* or subluxation* or luxation* or instability or loosen* or lysis or revision*).mp |
|  | **11** | 8 OR 9 OR 10 |
| **Adverse effect - medical devices** | **12** | complicat*.ti,ab. OR ae.fs. OR safe*.ti,ab. OR exp postoperative complications/ OR failure*.ti,ab. OR adverse.ti,ab. OR co.fs. OR failed.ti,ab. OR exp equipment failure/ OR removal.ti,ab. OR equipment safety/ OR problem*.ti,ab. OR side effect*.ti,ab. OR Harmful.ti,ab. OR Tolerated.ti,ab. OR loosen*.ti,ab. OR Intraoperative Complications/ OR migration.ti,ab. OR breakag*.ti,ab. OR discomfort.ti,ab. OR displacement.ti,ab. OR (detrimental adj2 effect*).ti,ab. OR untoward effects.ti,ab. |
| **Adverse effects - surgical interventions** | **13** | complication*.ti,ab. OR ae.fs. OR safe*.ti,ab. OR co.fs. OR postoperative complications/ |
|  | **14** | 12 OR 13 |
| **Combined** | **14** | 4 AND 7 AND 11 AND 14 |

| **Appendix 2. Patient characteristic of case series (n = 39)** | | | | | | | |
| --- | --- | --- | --- | --- | --- | --- | --- |
| **Study** | **Year** | **Country** | **Hips (n)** | **Patients (n)** | **Age (mean)** | **Males (n)** | **Mean follow up (yrs)** |
| Assi | 2018 | Lebanon | 30 | 26 | 55 | 12 | 4.3 |
| Bauchu | 2008 | France | 150 | 150 | 69 | 61 | 6.2 |
| Bouchet | 2011 | France | 105 | 105 | 76.6 | 45 | 2.3 |
| Boulat | 2019 | France | 33 | 33 | 74 | 4 | 3.7 |
| Combes | 2013 | France | 3474 | 3070 | 70 | 1290 | 7 |
| Chalmers | 2020 | USA | 86 | 80 | 69 | 23 | 3 |
| Chouteau | 2020 | Switzerland | 240 | 225 | 77.4 | 93 | 8.4 |
| Chughtai | 2016 | USA | 453 | 410 | 64 | 164 | 3 |
| Dubin | 2020 | USA | 664 | 664 | 61.7 | 356 | 2.1 |
| Epinette | 2015 | France | 143 | 136 | 70.6 | 49 | 4.45† |
| Epinette | 2016 | France | 321 | 321 | 55 | 167 | 2.7 |
| Ferreira | 2017 | France | 553 | 553 | 71.2 | 338 | 3* |
| Fessy | 2019 | France | 541 | 522 | 73.6 | 222 | 8.6 |
| Fresard | 2013 | France | 134 | 127 | 74 | 52 | 5.4 |
| Farizon | 1998 | France | 135 | 127 | 63 | 66 | 11.4 |
| Harwin | 2017 | USA | 249 | 249 | 66 | 103 | 3.3 |
| Kaiser | 2015 | Switzerland | 39 | 37 | 64 | 17 | 2* |
| Ko | 2016 | USA | 100 | 100 | n/a | n/a | 2.3 |
| Kreipke | 2019 | Denmark | 2277 | 2277 | 75.5 | 896 | 3 |
| Laurendon | 2018 | France | 100 | 93 | 71.8 | 50 | 10 |
| Leclercq | 2013 | France | 200 | 194 | 70 | 103 | 11 |
| Martz | 2016 | France | 40 | 31 | 44 | 23 | 9.7 |
| Massin | 2011 | France | 2408 | 2601 | 72 | 979 | 7.7 |
| Nam | 2019 | USA | 43 | 43 | 52.6 | 30 | 2 |
| Neri | 2016 | France | 212 | 174 | 53 | 104 | 25.3 |
| Nessler | 2020 | USA | 93 | 93 | 65.5 | 37 | 2.1 |
| Philippot | 2009 | France | 384 | 384 | 55.8 | 186 | 15 |
| Philippot | 2013 | France | 100 | 100 | 69.2 | 68 | 11.4 |
| Philippot | 2013 | France | 1960 | 1850 | 51 | n/a | 11 |
| Philippot | 2017 | France | 137 | 114 | 41 | 66 | 21.9 |
| Puch | 2016 | France | 119 | 105 | 49.9 | 79 | 11 |
| Prichett | 2017 | France | 160 | 160 | 43.5 | 76 | 11 |
| Rowan | 2017 | USA | 136 | 117 | 48.5 | 43 | 3.2 |
| Tabor-Jensen | 2020 | Denmark | 29 | 29 | 75 | 14 | 2 |
| Tabor-Jensen | 2020 | Denmark | 30 | 30 | 75 | 13 | 2 |
| Vermersch | 2015 | France | 104 | 100 | 73 | 40 | 6 |
| Vielpeau | 2011 | France | 437 | 389 | 61 | 236 | 16.5 |
| Vielpeau | 2011 | France | 231 | 221 | 74 | 104 | 5.2 |
| Vigdorchik | 2015 | USA | 485 | 450 | 66 | 262 | 2 |
| **Weighted totals** | n/a | n/a | **17,135** | **16,490** | **66.5** | **6,471 (39.2)** | **7.3** |
| n/a; not available, *; minimum follow up (yrs), †: maximum follow up (yrs) | | | | | | | |

| **Appendix 3. Summary of revision outcome data for case series (n = 39)** | | | | | | | |
| --- | --- | --- | --- | --- | --- | --- | --- |
| **Study** | **All-cause revision, n** | **Revised for instability, n** | **Revised for infection, n** | **Revised for fracture, n** | **Extra-articular**  **dislocation, n** | **IAPD, n** |  |
| Assi | 0 | 0 | 0 | 0 | 0 | 0 | |
| Bauchu | 3 | 0 | 0 | 1 | 0 | 0 | |
| Bouchet | 0 | 0 | 0 | 0 | 0 | 0 | |
| Boulat | 2 | 1 | 1 | 0 | 1 | 0 | |
| Combes | 37 | 1 | 0 | 5 | 23 | 7 | |
| Chalmers | 2 | 0 | 1 | 1 | 0 | 0 | |
| Chouteau | 6 | 0 | 4 | 1 | 0 | 0 | |
| Chughtai | 3 | 0 | 2 | 0 | 0 | 0 | |
| Dubin | 10 | 0 | 2 | 1 | 0 | 0 | |
| Epinette | 1 | 0 | 0 | 1 | 0 | 0 | |
| Epinette | 12 | 0 | 1 | 2 | 0 | 0 | |
| Ferreira | 2 | 0 | 1 | 1 | 0 | 0 | |
| Fessy | 13 | 0 | 6 | 0 | 0 | 0 | |
| Fresard | 3 | 0 | 0 | 0 | 0 | 0 | |
| Farizon | 5 | 1 | 0 | 0 | 2 | 0 | |
| Harwin | 3 | 0 | 1 | 1 | 0 | 0 | |
| Kaiser | 0 | 0 | 0 | 0 | 0 | 0 | |
| Ko | 0 | 0 | 0 | 0 | 1 | 0 | |
| Kreipke | 97 | 2 | 32 | 11 | 4 | 0 | |
| Laurendon | 4 | 0 | 0 | 0 | 0 | 0 | |
| Leclercq | 4 | 0 | 1 | 0 | 0 | 0 | |
| Martz | 0 | 0 | 0 | 0 | 0 | 0 | |
| Massin | 29 | 4 | 1 | 1 | 14 | 0 | |
| Nam | 0 | 0 | 0 | 0 | 0 | 0 | |
| Neri | 45 | 10 | 2 | 0 | 10 | 0 | |
| Nessler | 0 | 0 | 0 | 0 | 0 | 0 | |
| Philippot | 34 | 14 | 0 | 0 | 0 | 14 | |
| Philippot | 5 | 2 | 0 | 0 | 4 | 0 | |
| Philippot | 81 | 81 | 0 | 0 | 0 | 81 | |
| Philippot | 44 | 15 | 0 | 0 | 15 | 0 | |
| Puch | 4 | 0 | 0 | 0 | 0 | 0 | |
| Prichett | 6 | 0 | 2 | 0 | 0 | 0 | |
| Rowan | 27 | 0 | 0 | 0 | 0 | 0 | |
| Tabor-Jensen | 0 | 0 | 0 | 0 | 0 | 0 | |
| Tabor-Jensen | 1 | 1 | 0 | 0 | 1 | 0 | |
| Vermersch | 0 | 0 | 0 | 0 | 0 | 0 | |
| Vielpeau | 44 | 0 | 7 | 0 | 2 | 3 | |
| Vielpeau | 1 | 0 | 1 | 0 | 0 | 0 | |
| Vigdorchik | 3 | 0 | 0 | 1 | 0 | 0 | |
| **Totals (%)** | **531 (3.1)** | **132 (0.8)** | **65 (0.4)** | **57 (0.3)** | **77 (0.4)** | **105 (0.6)** | |
| IAPD; intra-prosthetic dislocation | | | | | | | |
